# FACES: A Deep-Learning-Based Parametric Model to Improve Rosacea Diagnoses

**DOI:** 10.3390/app13020970

**Published:** 2023-01-11

**Authors:** Seungman Park, Anna L. Chien, Beiyu Lin, Keva Li

**Affiliations:** 1 Department of Mechanical Engineering, University of Nevada, Las Vegas, NV 89154, USA; 2 Department of Dermatology, Johns Hopkins University School of Medicine, Baltimore, MD 21287, USA; 3 Department of Computer Science, University of Nevada, Las Vegas, NV 89154, USA; 4 Molecular and Cellular Biology, Johns Hopkins University, Baltimore, MD 21218, USA

**Keywords:** rosacea, deep learning, convolutional neural network (CNN), five accurate CNNs-based evaluation system (FACES), majority rule

## Abstract

Rosacea is a chronic inflammatory skin disorder that causes visible blood vessels and redness on the nose, chin, cheeks, and forehead. However, visual assessment, the current standard method used to identify rosacea, is often subjective among clinicians and results in high variation. Recent advances in artificial intelligence have allowed for the effective detection of various skin diseases with high accuracy and consistency. In this study, we develop a new methodology, coined “five accurate CNNs-based evaluation system (FACES)”, to identify and classify rosacea more efficiently. First, 19 CNN-based models that have been widely used for image classification were trained and tested via training and validation data sets. Next, the five best performing models were selected based on accuracy, which served as a weight value for FACES. At the same time, we also applied a majority rule to five selected models to detect rosacea. The results exhibited that the performance of FACES was superior to that of the five individual CNN-based models and the majority rule in terms of accuracy, sensitivity, specificity, and precision. In particular, the accuracy and sensitivity of FACES were the highest, and the specificity and precision were higher than most of the individual models. To improve the performance of our system, future studies must consider patient details, such as age, gender, and race, and perform comparison tests between our model system and clinicians.

## 1. Introduction

Rosacea is a chronic inflammatory skin disorder that causes visible blood vessels and redness on the human face [1]—a condition that affects more than 16 million Americans [2]. Globally, the prevalence of this skin condition is thought to range from 0.2% to 22% of the population in North America and Europe [3,4]. Rosacea is categorized into four types: erythematotelangiectatic, papulopustular, phymatous, and ocular rosacea [5,6]. However, it is often difficult to distinguish one type from another [7]. In addition, visual assessment of rosacea, the standard clinical diagnostic method, is often subjective among clinicians and results in high variation [8–10]. As such, in many cases, rosacea is misdiagnosed as other skin disorders, such as acne, eczema, and lupus, or vice versa due to their similarities in appearance [11].

Over the past decade, advances in computer-aided diagnosis and treatment using artificial intelligence have allowed for the detection of various skin diseases with high accuracy and consistency [12]. Furthermore, due to the COVID-19 pandemic and self-isolation in patients, telemedicine, which doesn’t require direct contact between clinicians and patients, has gained popularity [13]. The skin diseases for deep-learning-based classification are diverse, ranging from cancer to acne, eczema, psoriasis, etc. However, the majority of the studies have been focused on skin cancer research [14–22]. Hosny et al. employed a pre-trained deep learning network such as AlexNet to classify three different lesions (melanoma, common nevus, and atypical nevus) [23]. El-Khatib et al. developed a new, effective decision system to distinguish melanoma from a nevus, which combines several deep learning and machine learning models [24]. They employed five well-known convolutional neural network (CNN) models to develop a global classifier, which is the methodology used in this study, but they did not investigate numerous CNN models to find the five best. Moreover, they only tested a linear function to calculate the global index of decision and only tested the evaluation factor value (i.e., α = 0.7). Thomas et al. developed a new deep learning method for the effective detection of non-melanoma skin cancers, which are the most common skin cancers: basal cell carcinoma (BCC), squamous cell carcinoma (SCC), and intraepidermal carcinoma (IEC). Codella et al. used both deep learning and machine learning models to detect diverse skin lesions, including melanoma [25]. This study utilized the International Skin Imaging Collaboration (ISIC) database to classify 2624 dermoscopic images based on a sparse coding, deep residual network, and support vector machine. The results display the high-performance values of classification, with 93.1% accuracy, 92.8% specificity, and 94.9% sensitivity.

In addition to cancer, some efforts have been made to classify non-cancerous skin diseases through artificial intelligence, including psoriasis, atopic dermatitis, eczema, acne, hemangioma, onychomycosis, and so on [8,26,27]. Ramli et al. employed k-means clustering to classify acne lesions by collecting acne samples with different grades (mild, moderate, severe, and very severe) from 98 patients [28]. Aggarwal et al. trained TensorFlow Inception version 3 to recognize five dermatological diseases, including atopic dermatitis, acne, psoriasis, impetigo, and rosacea [29]. They measured six statistical parameters, such as sensitivity, specificity, PPV, NPN, MCC, and F1 score, with the application of data augmentation. Thomsen et al. adopted a pre-trained deep model, VGG-16, for the classification of multiple skin diseases (acne, rosacea, psoriasis, eczema, and cutaneous t-cell lymphoma) [15]. Goceri tested the performance of several deep learning models (U-Net, InceptionV3, ResNet, InceptionResNetV2, and VGGNet) to classify five common skin disorders: acne vulgaris, hemangioma, psoriasis, rosacea, and seborrheic dermatitis. The study showed that ResNet152 produces the highest accuracy [26].

Motivated by the recent success of artificial intelligence in detecting diverse skin disorders and diseases from clinical photos, we present a novel methodology integrating the existing CNN-based deep learning models to detect rosacea effectively. A total of 19 CNN-based models specialized in image classification were utilized and trained to recognize patterns from patients’ clinical images. The top five models were then selected in terms of accuracy, and subsequently, their accuracy values were used as weights for our generalized global model, coined “five accurate CNNs-based evaluation system (FACES)”. Using the five chosen models, we also applied a majority rule to classify rosacea, and its performance was compared with individual CNNs and FACES.

The Material and Methods section addresses the detailed methodology of FACES using different functions. Next, in the results section, we present the performance values for current and individual CNN models using four parameters. Finally, we discuss the potential limitations and future works in the discussion and conclusions sections.

## 2. Related Works

Currently, extremely few studies have been performed on automated identification only targeting rosacea using a deep learning or machine learning model, compared to other skin disorders. In addition, even though there are some studies, they usually include other skin lesions (Table 1), as mentioned above, rather than targeting rosacea alone, which can cause poor performance in terms of accuracy [26]. Recently, Binol et al. developed a new deep learning model, Ros-NET, to detect rosacea lesions by combining information from varying image scales and resolutions [14,30]. They estimated the Dice coefficient and false positive rate as a global measure using Ros-Net, whose results were compared with two well-known pre-trained deep learning models: Inception-ResNet-v2 and ResNet-101. However, most of these studies selected only a few models and compared their accuracy values to provide the best performing model. In this way, we could neglect the possibility that a better model might exist elsewhere. Hence, it is essential to develop a generalized global model encompassing numerous existing high-performance models.

## 3. Materials and Methods

### 3.1. Methodology for Skin Rosacea Detection Combining the Five Best Classifiers

**Figure 1.**
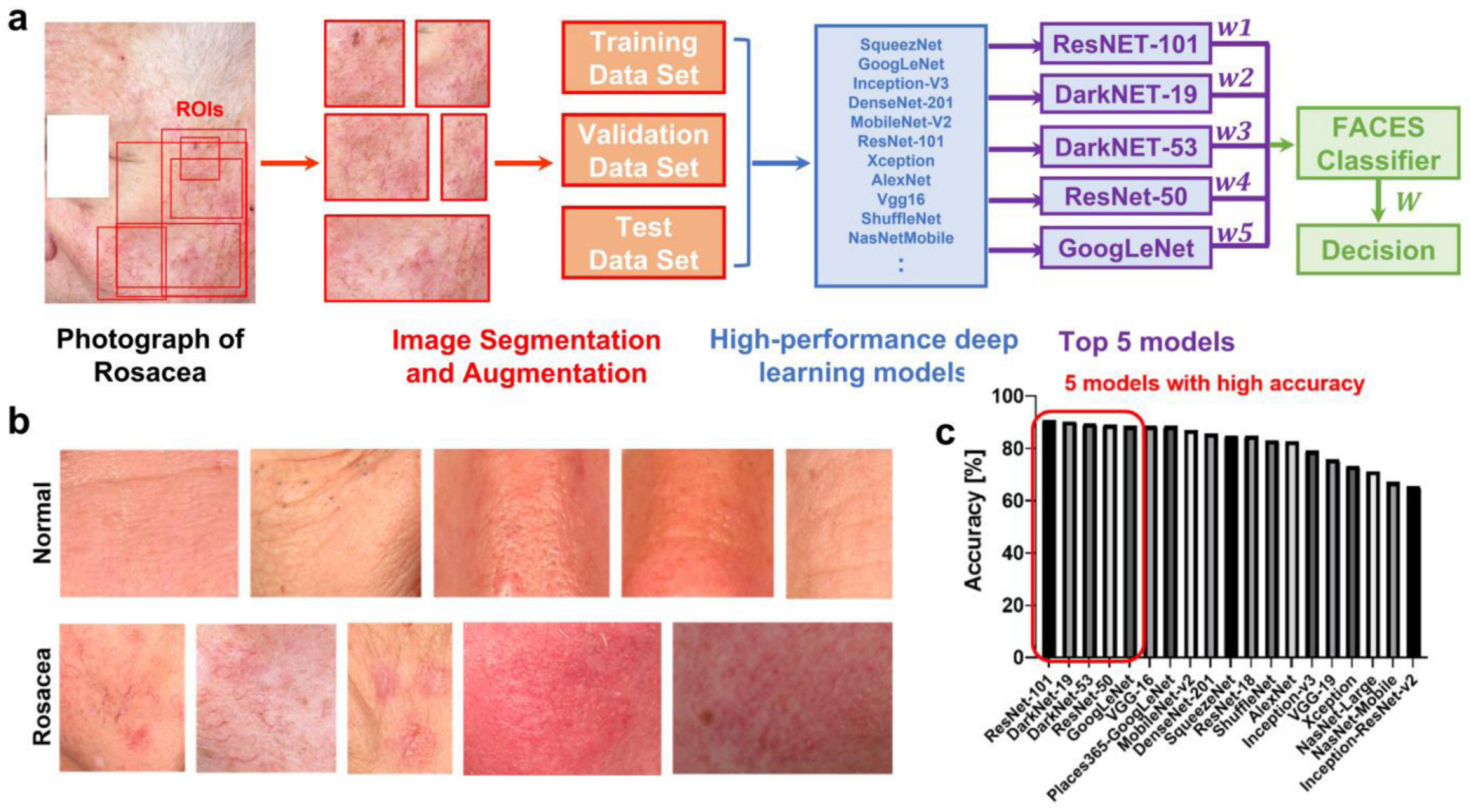
A five accurate CNNs-based evaluation system (FACES). (**a**) The workflow of FACES for identifying rosacea lesions. (**b**) Example images of normal and rosacea skin. (**c**) Accuracy values from 19 pre-trained CNNs.

The clinical images were obtained from 40 patients with rosacea and 59 control groups from Johns Hopkins University Hospital (Figure 1a, b). In order to improve the rosacea classification performance, we used multi-view clinical photos, which are photos of the same patient from different views. The high performance of multi-view, multi-modal, and integration approaches in machine learning and deep learning for image classification have been reported previously [32,33]. The images in the region of interest were segmented with different shapes and sizes, and subsequently, the segmented images were augmented by rotating and scaling (Figure 1a). The augmentation options included random reflections based on the *x*-axis (i.e., horizontal flipping), random rotation within the rate [−90, 90], and random rescaling within the range [1, 2]. The resolution of the images was approximately 15–20 pixels/mm. A total of 600 images (66% of the entire data set) for each group were used for training through the well-known 19 pre-trained CNNs (Figure 1c). A total of 200 images (22%) for each group were utilized for validation, and 110 images (12%) were utilized as test images. After training the CNN-based models, all models were tested to classify images using the validation data set. Consequently, the top 5 CNN-based models were selected based on accuracy to apply them to FACES (Figure 1c) as follows: ResNet-101 [34] (accuracy: 90.75%), DarkNet-19 [35] (90.25%), DarkNet-53 [36] (89.5%), ResNet-50 [37] (89.0%), and GoogleNet [38] (88.5%) (Table 2).

All of the selected 5 methods are CNN models that take a linear O(n) time, where n is the number of input pixels [39]. Floating point operations per second (FLOPS) reflects the computation complexity of CNN models. The FLOPSs for ResNet101, ResNet50, DarkNet53, DarkNet19, and GoogleNet are 7.6, 3.8, 53.6, 5.58, and 1.5 (billion), respectively. For our linear model, the total FLOPs are the summation of FLOPs of each selected method (i.e., 7.6 + 3.8 + 53.6 + 5.58 + 1.5 = 72.08 billion) because our methods are feedforward, traveling forward through the entire network.

Stochastic gradient descent with a momentum (SGDM) optimizer was used as a solver. Different values of initial learning rate (0.0001–0.01), validation frequency (1–10), max epochs (5–50), and mini-batch sizes (3–30) were tested to find optimum values for the highest accuracy because different CNN models exhibited different optimized values. In addition, L2 regularization was applied with a value of 0.0001 as another hyperparameter. The training time was highly dependent on the types and depths/layers of models ranging from 10 min to 7 h. All CNN models and FACES were run on a single CPU (Intel(R) Core(TM) i5–8265U CPU @ 1.80 GHz) and 8.00 GB RAM.

The accuracy values from the top 5 CNN-based models were used as weights in the FACES with the functions of 1st, 2nd, 3rd, and 4th degrees as follows:

1 $$W=\sum_{i=1}^{5}w_{i}^{n}d_{i}$$

2 $$W\geq \alpha W_{max}\text{and}W_{max}=\sum_{i=1}^{5}w_{i}^{n}$$

where n is 1, 2, 3, or 4 for linear, quadratic, cubic, and biquadratic functions, respectively; W is the FACES index of decision; $w_{i}$ is the weight (i.e., accuracy calculated in the validation phase for each classifier); $d_{i}$ is the individual decision, where 1, if the classifier, indicates rosacea; otherwise, 0; and α is the evaluation factor whose optimized value is determined using a parametric study testing α ranging from 0.1 to 0.9 with an increase of 0.1. It should be noted that FACES detects rosacea if threshold condition (2) is satisfied.

### 3.2. Methodology for Skin Rosacea Detection Using the Majority Rule

In addition to FACES, using the top 5 CNN-based models, the majority rule was applied to classify rosacea. In brief, among 5 selected CNN-based models, if the number of models indicating rosacea is equal to or greater than 3, the decision by the majority rule denotes rosacea. Otherwise, it indicates normal skin. All deep learning models used in this study were implemented using MATLAB R2021b.

### 3.3. Analysis of Four Performance Parameters

**Figure 2.**
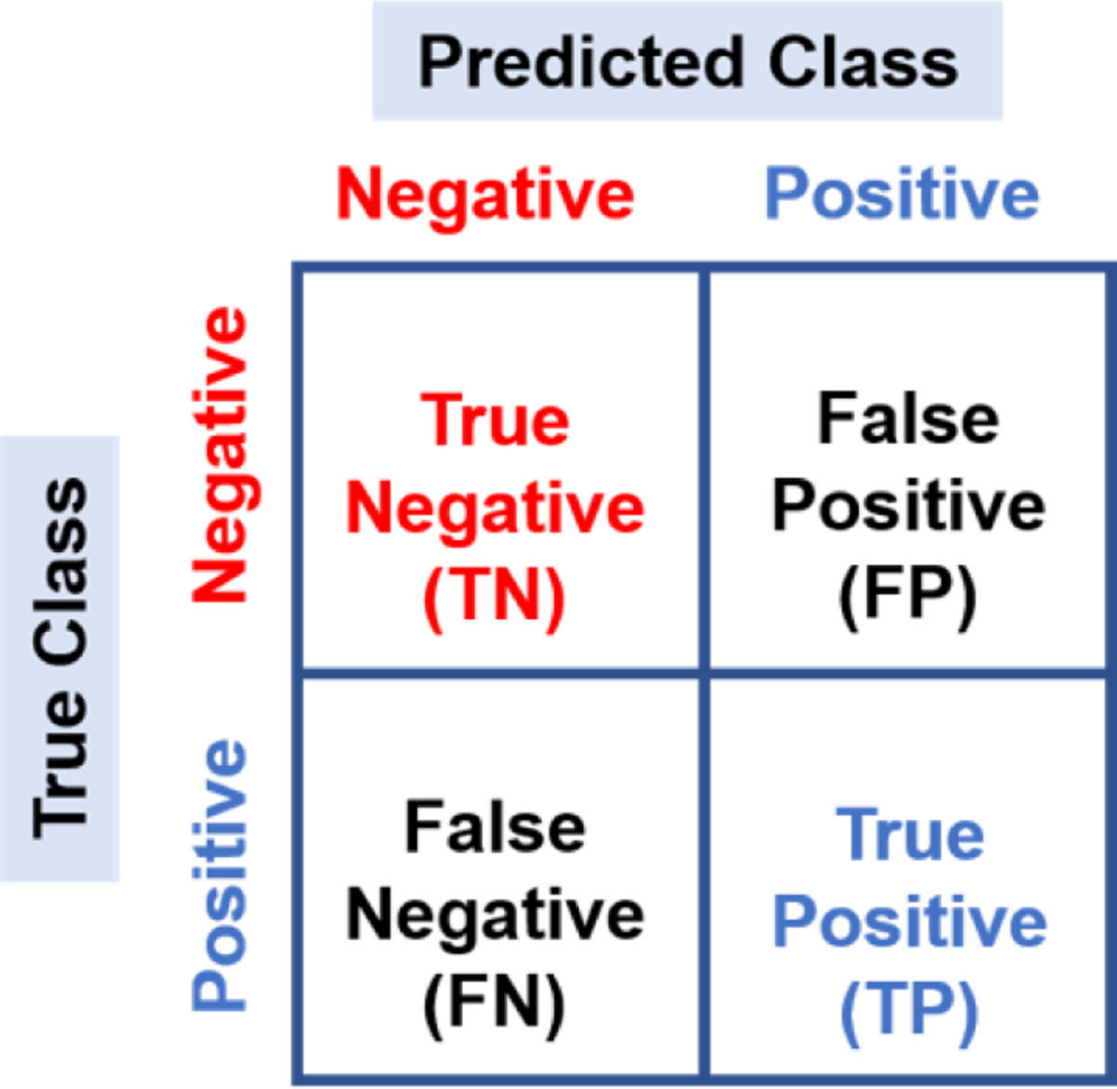
Schematic representation of the confusion matrix.

The accuracy, sensitivity, specificity, and precision of each model were calculated based on the confusion matrix containing true negative (TN), false negative (FN), true positive (TP), and false positive (FP) (Figure 2 and Table 3) to evaluate performance.

## 4. Results

**Figure 3.**
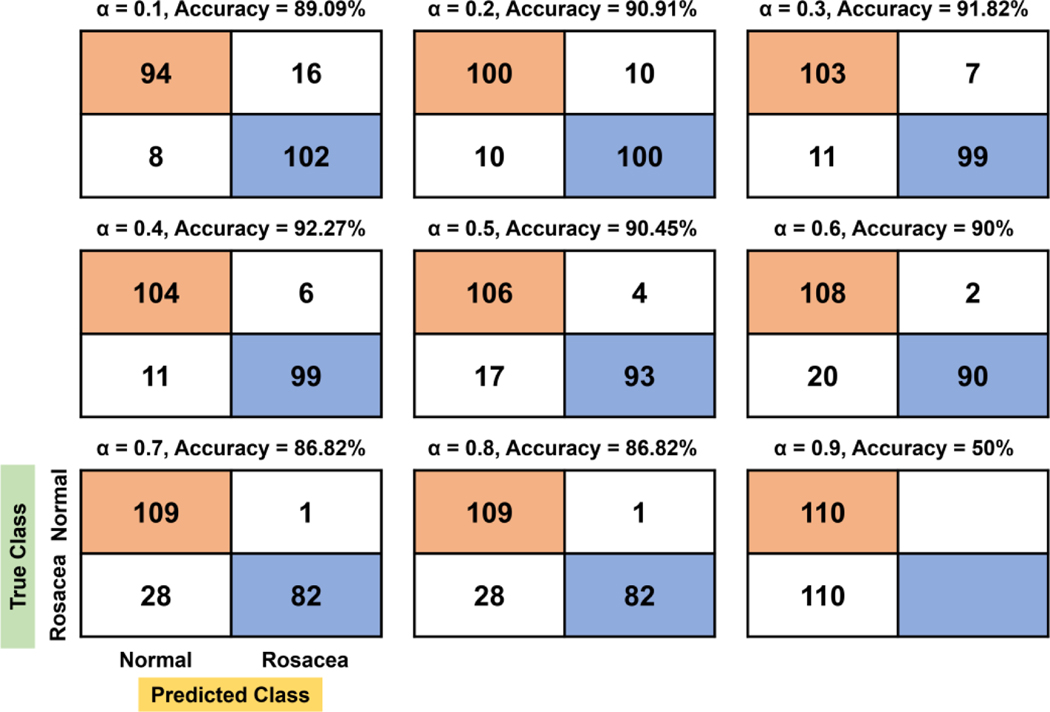
Confusion matrix and accuracy with respect to *α* for the linear function. In particular, the orange and blue colors denote true negative (TN) and true positive (TP), respectively.

**Figure 4.**
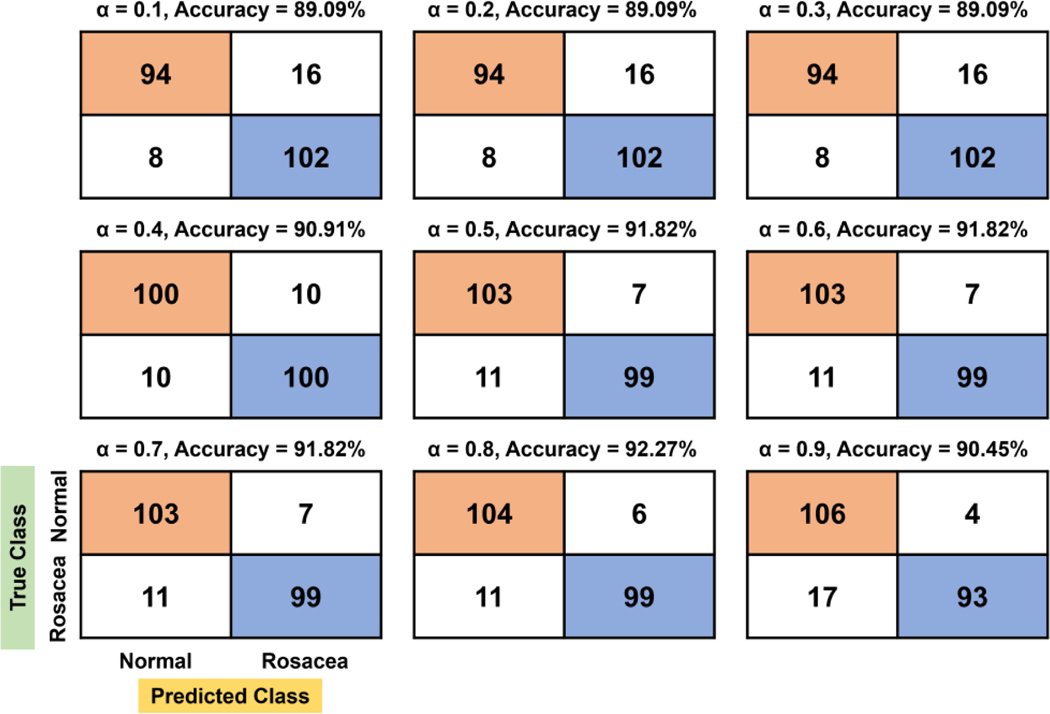
Confusion matrix and accuracy with respect to *α* for the quadratic function.

**Figure 5.**
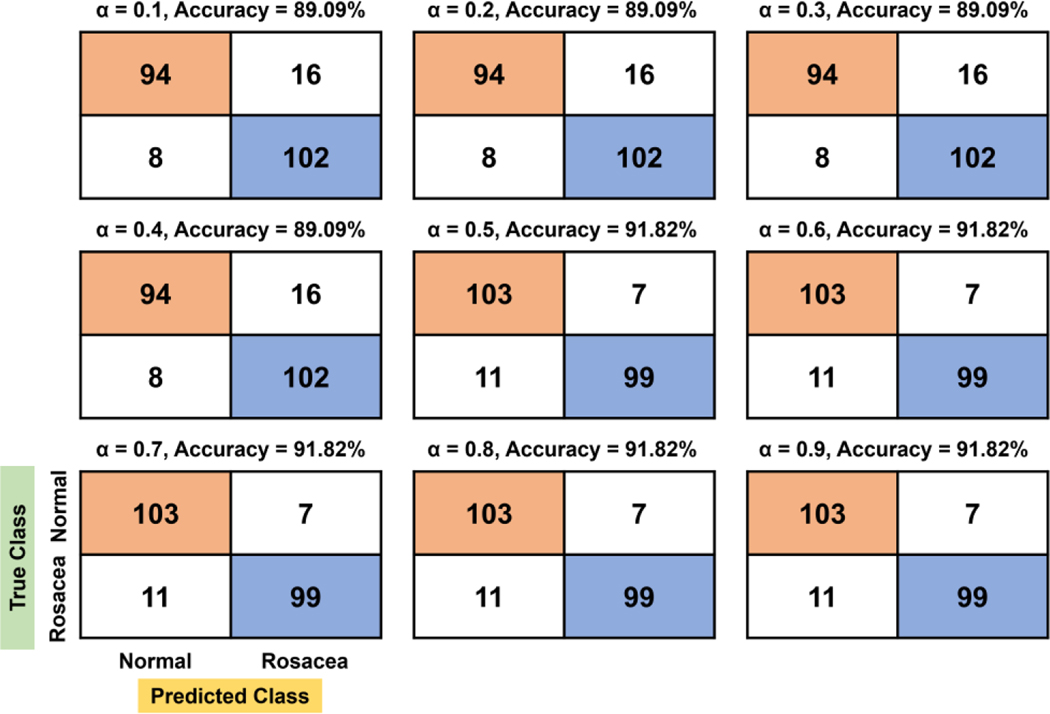
Confusion matrix and accuracy with respect to *α* for the cubic function.

**Figure 6.**
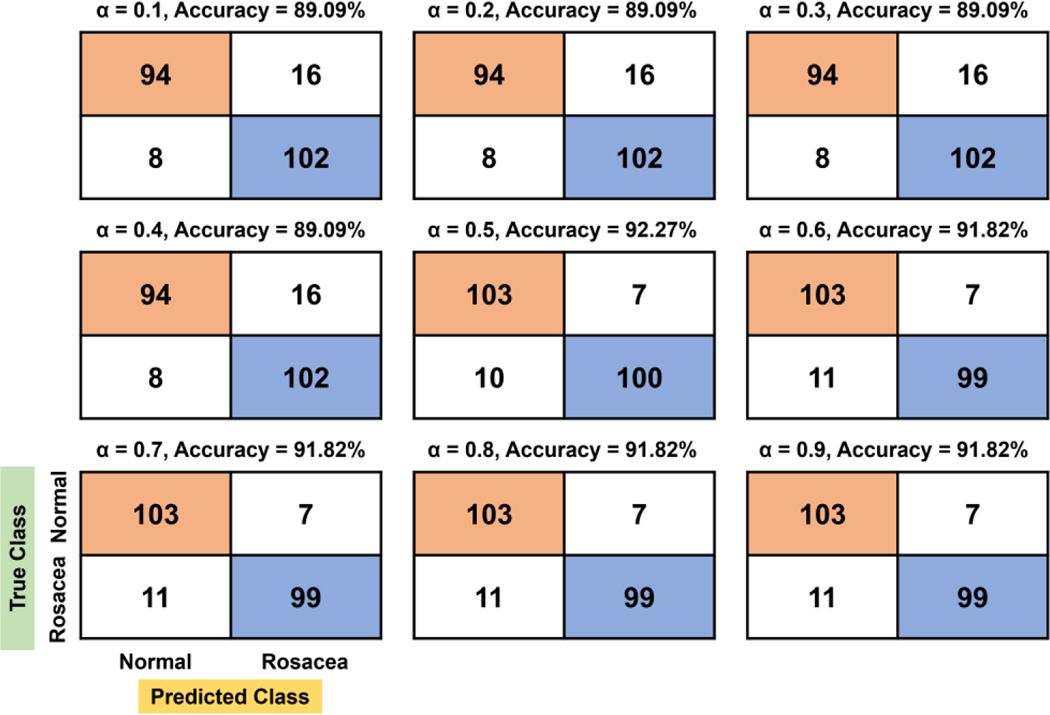
Confusion matrix and accuracy with respect to *α* for the biquadratic function.

We tested the effects of α ranging from 0.1 to 0.9 on the rosacea classification in the linear, quadratic, cubic, and biquadratic functions. The confusion matrices with respect to α are shown in Figures 3–6. For the linear function, the confusion matrix showed that true negative (TN) and false negative (FN) tend to increase as the evaluation factor increases, while true positive (TP) and false positive (FP) decrease (Figure 3). At the confusion matrix of highest accuracy (92.27%, α = 0.4), 10% (11/110) of rosacea and 5.45% (6/110) of normal skin are misinterpreted as normal skin and rosacea, respectively.

For the quadratic function, there are two stagnant areas showing constant TN, FN, TP, and FP: 0.1–0.3 and 0.5–0.7 (Figure 4). The best accuracy (92.27%) value is found to be at 0.8 of α, where 10% (11/110) of rosacea and 5.45% (6/110) of normal skin are misclassified as normal skin and rosacea, respectively. Overall, TN and FN tend to increase more gradually with the increasing evaluation factor compared to the linear function, while TP and FP tend to decrease.

For the cubic function, there are two large stagnant regions: the first region ranging from 0.1 to 0.4 and the second region ranging from 0.5 to 0.9 (Figure 5). The confusion matrix showed constant accuracy for each of the first and second stagnant areas at 89.09% and 92.27%, respectively. It should be noted that the TP of the first region is higher than that of the second region, while TN is the opposite, showing a lower value in the first region. At the region of highest accuracy (91.82%), 10% (11/110) of rosacea and 6.36% (7/110) of normal skin are misinterpreted as normal skin and rosacea, respectively.

For the biquadratic function, a single α (0.5) shows the best accuracy (92.27%), while two stagnant regions (0.1–0.4 and 0.6–0.9) reveal accuracy values at 89.09% and 91.82%, respectively (Figure 6). At the confusion matrix of highest accuracy (92.27%, α = 0.5), 9.09% (10/110) of rosacea and 5.45% (6/110) of normal skin are misinterpreted as normal skin and rosacea, respectively.

**Figure 7.**
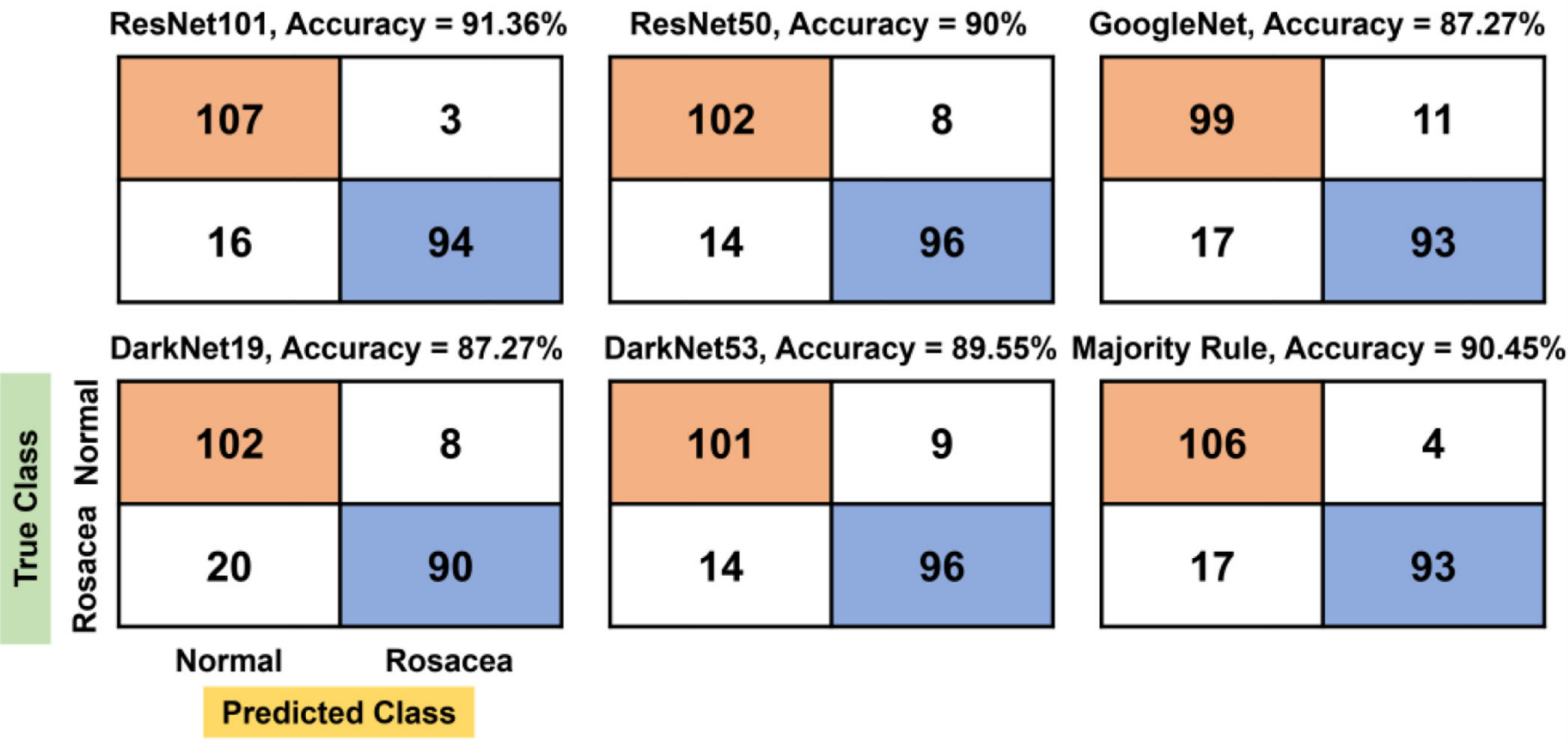
Confusion matrix and accuracy by the majority rule and other individual CNN models.

The confusion matrix created by the majority rule yields 90.45% accuracy as well as TN (106), which is the second largest, following ResNet-101 (91.36% for accuracy and 107 for TN) (Figure 7). In addition, in the majority rule, 15.45% (17/110) of rosacea and 3.64% (4/110) of normal skin are misinterpreted as normal skin and rosacea, respectively.

**Figure 8.**
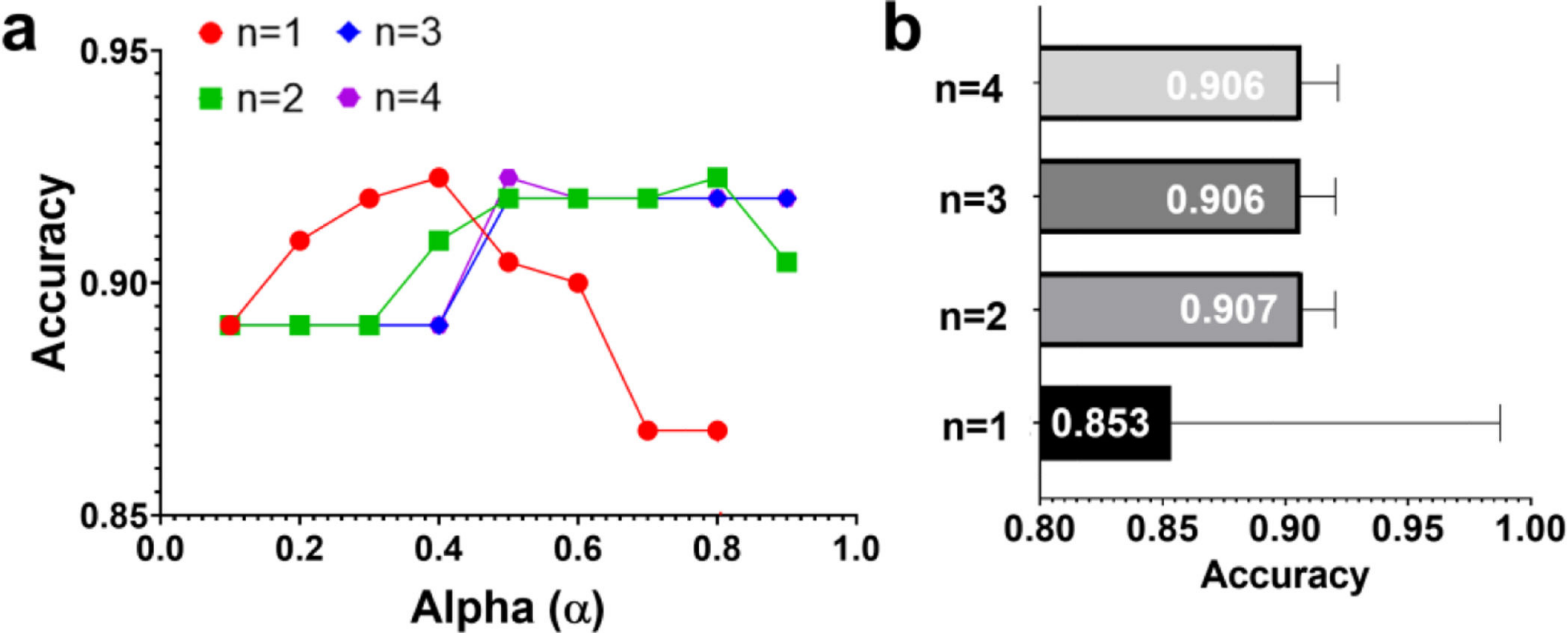
Parametric analysis in different functions with respect to the evaluation factor (α) of the FACES. (**a**) Effects of α on accuracy values with different degrees of polynomial order (n). (**b**) Average accuracy values of each function: linear (n = 1), quadratic (n = 2), cubic (n = 3), and biquadratic (n = 4) functions.

On the basis of the results from the confusion matrices, accuracy is shown as a function of α for each function. The results show that there is a significant impact of the evaluation factor on the accuracy, but their distribution depends highly on the degree of function (Figure 8a). To be more specific, for the linear function (n = 1), accuracy tends to increase up to 0.4 of α continually but decreases after that. For the quadratic, cubic, and biquadratic functions (n = 2, 3, and 4), the accuracy drastically increases to 0.3 or 0.4 of α and remains almost constant after 0.5 of α. The average accuracy values are 0.853, 0.907, 0.906, and 0.906 for the linear, quadratic, cubic, and biquadratic functions, respectively. The highest accuracy is 92.27% (α = 0.4), 92.27% (α = 0.8), 91.82% (α ≥ 0.5), and 92.27% (α = 0.5) for the linear, quadratic, cubic, and biquadratic functions, respectively. When both the highest and average accuracy are considered, the quadratic function can be selected as the best performing function. Noticeably, all four performance parameters driven by FACES are at least greater than or equal to 0.9, while those by other models are not.

**Figure 9.**
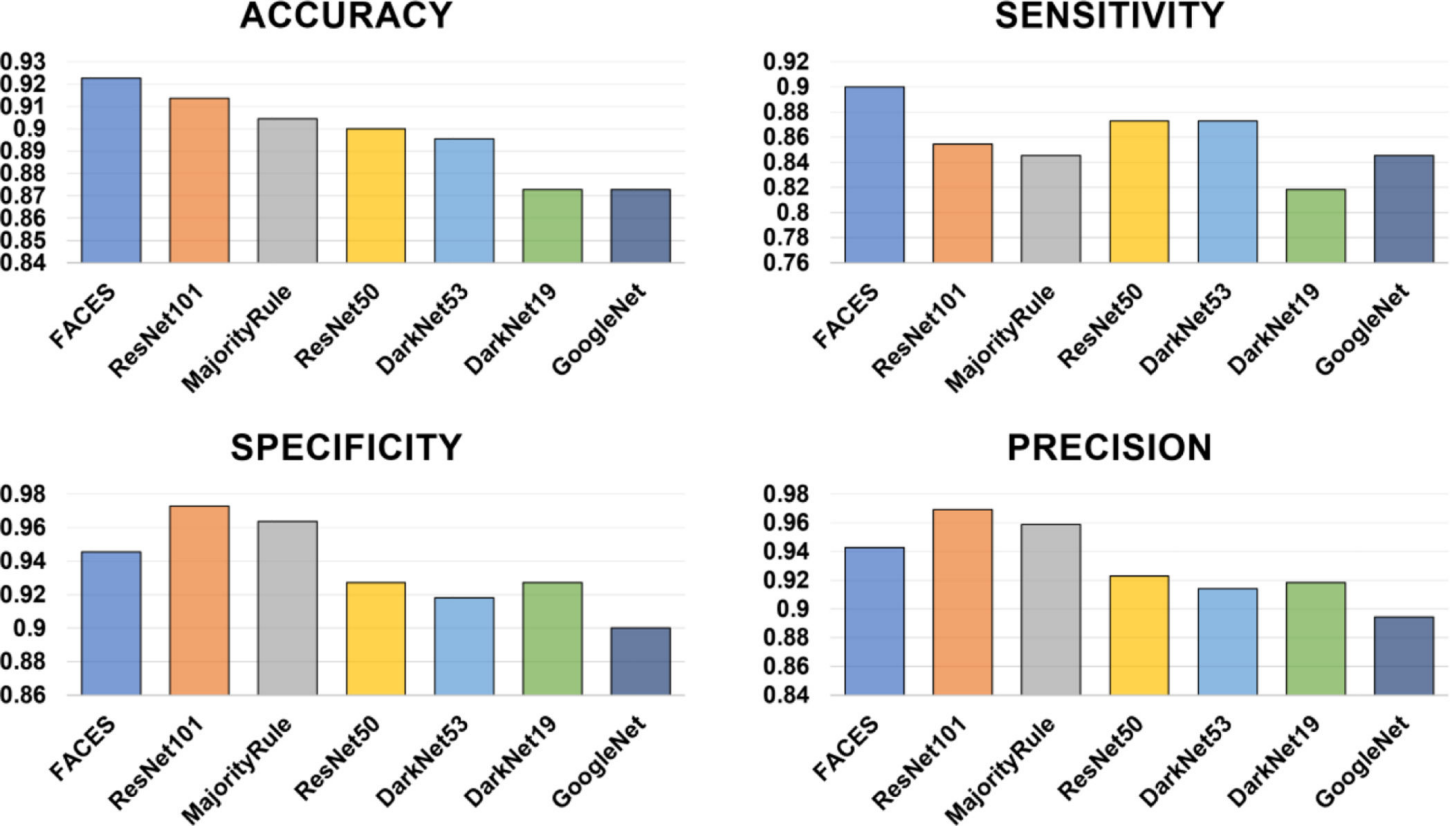
Comparison of performance of FACES with that of individual CNNs and the majority rule in the testing phase.

We compared the performance of the FACES with that of individual CNN-based models and the majority rule. The results show that FACES displays the best performance, revealing the highest accuracy (92.27%), followed by ResNet-101 (91.36%) and the majority rule (90.45%), as well as the highest sensitivity (0.90), followed by ResNet50 (0.873) and DarkNet53 (0.873) (Figure 9). However, FACES’s specificity (0.945) and precision (0.943) are the third highest, following ResNet101 (0.973 for specificity and 0.969 for precision) and the majority rule (0.964 and 0.959).

## 5. Discussion

Different individual CNN models have different performances for image classification and identification. In this study, by combining the results of these models, we developed a better system with higher accuracy to detect rosacea. A comparison between the results of our proposed system and the results produced by other studies is shown in Table 4. Our proposed system shows better performance (92.27%) than that of the study with the highest accuracy (90.2%, Ros-Net).

For rosacea identification and classification, we utilized clinical photos taken by a digital camera covering the whole face. However, a single photo might not cover the proportion of the skin lesion, thereby hardly representing or even distorting the features of rosacea. To overcome such limitations, we took photos from at least three different angles (i.e., multi-view learning), which contain the whole lesion of rosacea. Moreover, clinical photos were segmented and augmented to integrate different-scaled images to provide both microscopic and macroscopic characteristics of rosacea to improve the performance of FACES.

There are still limitations to be addressed and factors to be explored in the future to improve FACES’s ability to detect rosacea. First, we did not compare the performance between FACES and experienced dermatologists to validate our method. Hence, comparison tests are required to warrant using our detection tool in clinical settings. In addition, we did not consider several important parameters, such as race, age, or gender, which can be highly associated with the occurrence of rosacea, thus, affecting FACES’s classification abilities. Early studies demonstrated that rosacea is an age-related disease that occurs more frequently at an older age, particularly above the age of 65 years [40,41]. Moreover, women under the age of 49 were found to be more affected by rosacea, while rosacea was more prevalent in men over 50 [40]. Rosacea’s prevalence is also highly dependent on race. Prior research illustrated that Hispanics and Latinos are more susceptible to rosacea compared to African Americans or Asians [42]. Taken together, these limitations, as well as factors such as age, gender, or race, should be considered in future studies to enhance the performance of artificial intelligence. Such an approach will shed light on new effective diagnoses and rosacea treatment in clinical practice.

## 6. Conclusions

In this study, we developed a new decision system based on high-performance CNN-based pre-trained models for the detection of rosacea—FACES. FACES outperformed other individual models, showing greater accuracy and sensitivity than each individual classifier. In addition, FACES performed well in terms of specificity and precision. It is expected that the current workflow can be extended and applied to other types of skin diseases in future studies. However, diverse rosacea-related factors need to be systematically considered as a deep learning parameter in future studies to improve rosacea identification.

## Supplementary Material

Supplementary Material

## Figures and Tables

**Table 1.** Previous related studies for the identification of rosacea.

Method	Skin Lesions	References
U-Net, VGGNet, Inception-v3, InceptionResNet-v2, and ResNet	Rosacea, acne vulgaris, hemangioma, psoriasis, and seborrheic dermatitis	Goceri [26]
Inception-v3	Rosacea, acne, atopic dermatitis, psoriasis, and impetigo	Aggarwal [29]
DenseNet201	Rosacea, acne vulgaris, hemangioma, psoriasis, and seborrheic dermatitis	Goceri [31]
VGG-16	Rosacea, acne, psoriasis, eczema, and cutaneous t-cell lymphoma	Thomsen et al. [15]
Ros-Net	Rosacea	Binol et al. [14]
Inception-ResNet-v2	Rosacea	Binol et al. [30]

**Table 2.** Properties of the 5 used models for FACES.

Network	Depth	Size	Parameters (Millions)	Image Input Size
ResNet-101	101	167 MB	44.6	224 by 224
DarkNet-19	19	78 MB	20.8	256 by 256
DarkNet-53	53	155 MB	41.6	256 by 256
ResNet-50	50	96 MB	25.6	224 by 224
GoogleNet	22	27 MB	7.0	224 by 224

**Table 3.** Parameters of performance used in this study.

Performance Parameter	Formula
Accuracy	(TN + TP)/(TN + FP + FN + TP)
Sensitivity	TP/(TP + FN)
Specificity	TN/(TN + FP)
Precision	TP/(FP + TP)

**Table 4.** Comparison of accuracy values for the rosacea detection.

Method	Accuracy	References

FACES	92.27%	Current study

ResNet-50	79%	[26]

Ros-Net		
• Overlapping image patches	90.2%	[14,30]
• Non-overlapping image patches	88%	

DenseNet201	87.81%	[31]

VGG-16	88.64%	[15]

## Data Availability

The datasets presented in this article are not readily available because the datasets consist of clinical images of patients with rosacea. The source code for the FACES classification algorithm is available in the Supplementary Data.

## Abbreviation

FACES: five accurate CNNs-based evaluation system
BCC: basal cell carcinoma
SCC: squamous cell carcinoma
IEC: intraepidermal carcinoma
TN: true negative
FN: false negative
TP: true positive
FP: false positive
