# Supplementary material for: FACES: A Deep-Learning-Based Parametric Model to Improve Rosacea Diagnoses
Source: Appl Sci (Basel). Author manuscript; Available in PMC 2024 Jan 26. (PMC10817774; doi:10.3390/app13020970)
Supplement: Supplementary Material [file NIHMS1871776-supplement-Supplementary_Material.pdf]

```
%% Test images using the trained networks

% clear all;
% clc;

imdsTest =
imageDatastore("C:\Users\smpar\Desktop\SkinPhoto\DeepLearning\Cr
opped
photos3_CutOff_Outlier\TestImages\Images","IncludeSubfolders",tr
ue,"LabelSource","foldernames");

num=5; % Number of CNNs

%% (1). Classify the images_ResNet101
testds=augmentedImageDatastore([224 224 3], imdsTest);

% Make predictions
[preds1, probs1]=classify(ResNet101_Skin_Trained,testds);

% Compare with reality
truetest=imdsTest.Labels;
Accuracy_ResNet101=nnz(preds1 == truetest)/numel(preds1)
w1=ResNet101_Skin_TrainedInfo.FinalValidationAccuracy/100;

% Calculate d value
n=length(truetest);
for j=1:1:n
    if preds1(j) == "Rosacea"
        d1(j)=1;
    else
        d1(j)=0;
    end
end

% View confusion matrix
figure(1)
subplot(2,3,1)
confusionchart(truetest,preds1);
title("ResNet101,
Accuracy="+num2str(round(Accuracy_ResNet101,4)*100)+"%");

%% (2). Classify the images_ResNet50
testds=augmentedImageDatastore([224 224 3], imdsTest);

% Make predictions
[preds2, probs2]=classify(ResNet50_Skin_Trained,testds);
```

```
% Compare with reality
truetest=imdsTest.Labels;
Accuracy_ResNet50=nnz(preds2 == truetest)/numel(preds2)
w2=ResNet50_Skin_TrainedInfo.FinalValidationAccuracy/100;
% w3=Accuracy_ResNet50;

% Calculate d value
n=length(truetest);
for j=1:1:n
    if preds2(j) == "Rosacea"
        d2(j)=1;
    else
        d2(j)=0;
    end
end

% View confusion matrix
subplot(2,3,2)
confusionchart(truetest,preds2);
title("ResNet50,
Accuracy="+num2str(round(Accuracy_ResNet50,4)*100)+"%");

%% (3). Classify the images_GoogleNet
testds=augmentedImageDatastore([224 224 3], imdsTest);

% Make predictions
[preds3, probs3]=classify(GoogleNet_Skin_Trained,testds);

% Compare with reality
truetest=imdsTest.Labels;
Accuracy_GoogleNet=nnz(preds3 == truetest)/numel(preds3)
w3=GoogleNet_Skin_TrainedInfo.FinalValidationAccuracy/100;
% w1=Accuracy_GoogleNet;

% Calculate d value
n=length(truetest);
for j=1:1:n
    if preds1(j) == "RosaceaImages"
        d3(j)=1;
    else
        d3(j)=0;
    end
end

% View confusion matrix
subplot(2,3,3)
confusionchart(truetest,preds3);
```

```
title("GoogleNet,
Accuracy="+num2str(round(Accuracy_GoogleNet,4)*100)+"%");

%% (4). Classify the images_DarkNet19
testds=augmentedImageDatastore([256 256 3], imdsTest);

% Make predictions
[preds4, probs4]=classify(DarkNet19_Skin_Trained,testds);

% Compare with reality
truetest=imdsTest.Labels;
Accuracy_DarkNet19=nnz(preds4 == truetest)/numel(preds4)
w4=DarkNet19_Skin_TrainedInfo.FinalValidationAccuracy/100;
% w5=Accuracy_DarkNet53;

% Calculate d value
n=length(truetest);
for j=1:1:n
    if preds4(j) == "Rosacea"
        d4(j)=1;
    else
        d4(j)=0;
    end
end

% View confusion matrix
subplot(2,3,4)
confusionchart(truetest,preds4);
title("DarkNet19,
Accuracy="+num2str(round(Accuracy_DarkNet19,4)*100)+"%");

%% (5). Classify the images_DarkNet53
testds=augmentedImageDatastore([256 256 3], imdsTest);

% Make predictions
[preds5, probs5]=classify(DarkNet53_Skin_Trained,testds);

% Compare with reality
truetest=imdsTest.Labels;
Accuracy_DarkNet53=nnz(preds5 == truetest)/numel(preds5)
w5=DarkNet53_Skin_TrainedInfo.FinalValidationAccuracy/100;
% w5=Accuracy_DarkNet53;

% Calculate d value
n=length(truetest);
for j=1:1:n
    if preds5(j) == "Rosacea"
```

```
        d5(j)=1;
    else
        d5(j)=0;
    end
end

% View confusion matrix
subplot(2,3,5)
confusionchart(truetest,preds5);
title("DarkNet53,
Accuracy="+num2str(round(Accuracy_DarkNet53,4)*100)+"%");

%% (6). Classify the images_Majority Rule

for k=1:1:n
    M(k)=d1(k)+d2(k)+d3(k)+d4(k)+d5(k);
    if M(k) >= round(num/2)
        preds_M(k,1) = "Rosacea";
    else
        preds_M(k,1) = "Control";
    end
end
preds_M=categorical(preds_M);

% Compare with reality
truetest=imdsTest.Labels;
Accuracy_MajorityRule=nnz(preds_M == truetest)/numel(preds_M)

% View confusion matrix
subplot(2,3,6)
confusionchart(truetest,preds_M);
title("Majority Rule,
Accuracy="+num2str(round(Accuracy_MajorityRule,4)*100)+"%");

%% (7). Classify the images using FACES
alpha=[0.1 0.2 0.3 0.4 0.5 0.6 0.7 0.8 0.9]; % Experimentally
measured (Evaluation factor)

num_alpha=length(alpha);
% Accuracy_FACES=zeros(1,num_alpha)'; % Use allocate spaces
using Zeros!! (Very important!!!)
for m=1:1:num_alpha
%     w_max=w1+w2+w3+w4+w5;
%     w_max=(w1.^2+w2.^2+w3.^2+w4.^2+w5.^2)^0.5;
    w_max=(w1.^4+w2.^4+w3.^4+w4.^4+w5.^4)^(1/4);
    w_threshold(m)=w_max*alpha(m);
```

```
    % Evaluate whether or not this is rosacea
    for k=1:1:n
%         W(k)=w1*d1(k)+w2*d2(k)+w3*d3(k)+w4*d4(k)+w5*d5(k);
%
W(k)=w1.^2*d1(k)+w2.^2*d2(k)+w3.^2*d3(k)+w4.^2*d4(k)+w5.^2*d5(k)
;

W(k)=w1.^4*d1(k)+w2.^4*d2(k)+w3.^4*d3(k)+w4.^4*d4(k)+w5.^4*d5(k)
;
        if W(k) >= w_threshold(m)
            preds_global(k,1) = "Rosacea";
        else
            preds_global(k,1) = "Control";
        end
    end
    preds_global=categorical(preds_global);

    % Compare with reality
    truetest=imdsTest.Labels;
    Accuracy_GlobalDecison(m)=nnz(preds_global ==
truetest)/numel(preds_global)
    Accuracy_GlobalDecison=Accuracy_GlobalDecison'

    % View confusion matrix
    figure(2)
    subplot(3,3,m)
    confusionchart(truetest,preds_global);
    title("FACES Decision (alpha="+ num2str(alpha(m))+"),
Accuracy="+num2str(round(Accuracy_GlobalDecison(m),4)*100)+"%");
end
```
